# Sex-related differences in the association between migraine, COVID-19, and long COVID: a population-based cohort

**DOI:** 10.3389/fneur.2025.1547893

**Published:** 2025-03-21

**Authors:** Linda Al-Hassany, Antoinette MaassenVanDenBrink, Tobias Kurth

**Affiliations:** ^1^Erasmus MC, University Medical Center Rotterdam, Division of Vascular Medicine and Pharmacology, Department of Internal Medicine, Rotterdam, Netherlands; ^2^Institute of Public Health, Charité—Universitätsmedizin Berlin, Berlin, Germany

**Keywords:** migraine, COVID-19, long COVID, headache, anosmia, memory, concentration, sex differences

## Abstract

**Background:**

Coronavirus disease 2019 (COVID-19), caused by the SARS-CoV-2 virus, placed unprecedented pressure on public health systems due to its mortality and global panic—and later due to long COVID challenges. One of these long COVID symptoms, headache, often resembles migraine-like features. Migraine shares similarities with COVID-19 and long COVID, yet the influence of sex is understudied. Our primary objective was to study the interrelationship between COVID-19 and migraine prevalence, while considering sex differences. The secondary objective was to examine how long COVID symptoms (headache, anosmia, memory, and concentration problems) affect males and females with and without COVID-19 and migraine.

**Methods:**

All analyses were conducted using Lifelines, a prospective cohort study in the northern Netherlands. Baseline characteristics (2006–2014), self-reported migraine diagnoses (until 2021), and questionnaires on COVID-19 and long COVID symptoms (2020–2022) were collected. Logistic regression analyses were conducted to study the association between lifetime migraine and current SARS-CoV-2 infections while adjusting for age, sex, diet, educational attainment, activity, and smoking. Descriptive and sex-stratified analyses were conducted on long COVID symptoms.

**Results:**

A total of 150,507 individuals were included, of which 29,680 (19.7%) reported migraine and 120,827 (80.3%) not. A total of 1,867 individuals with migraine [6.3% of individuals with migraine, 44.0 years (IQR 36.1–50.3)] and 6,797 individuals without migraine [5.6% of individuals without migraine, 44.4 years (IQR 35.3–52.2)] reported to be SARS-CoV-2 infected. The majority of individuals with migraine consisted of females (77.0% of those with migraine vs. 54.0% of those without migraine). The adjusted odds of having SARS-CoV-2 infections was 6.3% higher among those with (a history of) migraine compared with individuals without migraine in the logistic regression model (OR = 1.06, 95% CI 1.01–1.12). A slightly higher OR was observed in females (OR = 1.08, 95% CI 1.02–1.15), and the association was not apparent in males (OR = 1.00, 95% CI 0.88–1.12). Secondary analyses revealed that individuals with both migraine and COVID-19, and females in particular, were the most frequently bothered by long COVID symptoms headache, anosmia, concentration, and memory problems. Individuals with none of these diseases were the least bothered.

**Conclusions:**

Individuals with migraine, especially females, are slightly more likely to report and/or contract COVID-19. Those with both conditions report long COVID symptoms more frequently, suggesting a shared vulnerability or pathophysiology. This may indicate the need for clinical surveillance of migraine patients recovering from COVID-19.

## Introduction

Coronavirus disease 2019 (COVID-19), caused by severe acute respiratory syndrome coronavirus 2 (SARS-CoV-2), has put unprecedented pressure on public health systems and led to over 775 million cumulative cases and 7.1 million deaths globally (1). Great advances have been made in the diagnostics of COVID-19 (2) and the development of life-saving vaccines (3)—leading to a transition of the pandemic disease to a more manageable disease. Whereas, acute respiratory symptoms and lung failure have dominated the clinical manifestations of COVID-19 in the acute phase (4), a substantial number of individuals continue to suffer from heterogeneous prolonged symptoms or so-called “long COVID.” These symptoms range from neurological symptoms and cardiac abnormalities to chronic fatigue (5). One of these most prevalent neurological symptoms is headache, which could manifest as either a new headache or aggravation (in frequency or severity) of preexisting primary headache, including migraine (6). Interestingly, migraine-like features of especially persisting long COVID headaches have been reported in several studies (7–9). Further similarities include the incidence of long COVID headaches and the prevalence of migraine, which both are increased in females (10, 11), and a peak in diagnoses of both diseases until the age of fifty (12). In contrast to the gain in function in the trigeminal system, i.e., headache, another neurologic manifestation of long COVID is a loss of function in the olfactory system, i.e., anosmia (13), which also seems to differ between sexes and age categories (14) and to be increased in migraine patients (15). Further, neuropsychiatric symptoms, including (objectively measured) cognitive impairment and “brain fog,” have been associated with chronic post-COVID-19, especially among females (16–18). Similar symptoms have been described for migraine, even in the interictal phase (19). Notably, these similarities between long COVID (as a symptom of COVID-19) and migraine are not observed in the clinical manifestations of acute SARS-CoV-2 infections. Indeed, retrospective observations indicate that males are rather more likely to exhibit enhanced COVID-19 severity and mortality than females—probably also due to sex differences in the immune response (20, 21).

The interrelationships of migraine, COVID-19, and the long COVID symptoms of headache, anosmia, and memory or concentration problems while accounting for sex differences are understudied. Indeed, the importance of sex and gender, including hormonal influences, in migraine is increasingly being recognized, especially with regard to the prevalence and pathophysiology (22, 23). Yet, there has been a lack in the application of sex-specific methodologies in COVID-19 research, contributing to knowledge gaps on the diagnosis, including the recognition of individuals who are at risk and treatment of long-term symptoms, which have a substantial influence on the quality of life (24, 25). The overlapping clinical symptoms and the enormous socioeconomic burden of migraine, COVID-19, and long COVID-19 probed us to study the influence of sex in their association in a large prospective population-based setting. Thus, our primary objective was to study the interrelationship of COVID-19 and migraine prevalence, accounting for sex differences. Our secondary objective was to study the extent to which individuals, both male and female, with and without COVID-19 and migraine, had long COVID symptoms, i.e., headache, anosmia, and memory and concentration problems.

## Method

### Study population

All analyses were performed within Lifelines, a multidisciplinary prospective population-based cohort study consisting of a total number of 167,729 persons, representing ~10% of the total population in the three northern provinces of the Netherlands. The Lifelines cohort was established in 2006 and designed to enhance our understanding of the complex interactions between genetics, lifestyle, and environment in relation to health (including aging) and (chronic) disease. No specific inclusion criteria were applied, and the risk of selection bias has been demonstrated to be low (26). Individuals were excluded from participation in case they (i) had a terminal illness (life expectancy of < 5 years), (ii) were incapacitated, e.g., due to a severe mental illness, (iii) were unable to visit their general practitioner, (iv) were unable to complete the questionnaires, or (v) had insufficient knowledge of the Dutch language. The collection of baseline characteristics took place between 2006 and 2014. Follow-up procedures included the completion of (digital) questionnaires sent approximately every 1.5–2.5 years.

The current study is based on an add-on to the ongoing observational cohort, i.e., the Lifelines COVID-19 cohort study (27). Questionnaires containing questions on COVID-19 diagnoses, symptoms, comorbidities, and mental aspects, among others, were sent to all Lifelines participants aged ≥18 years with a known email address. These questionnaires were initially sent out weekly, but they were sent out every 2 weeks from June 2020 and every month from August 2020.

The Lifelines Cohort Study, including the COVID-19 add-on study, is conducted according to the principles of the Declaration of Helsinki and approved by the Medical Ethics Committee of the University Medical Center Groningen, the Netherlands (reference numbers METc 2007/152 and METc 2019/571; approval number NL17981.042.07). The overall design and rationale of the cohort have been described in detail elsewhere (28, 29).

### Data collection

All individuals aged ≥18 years who signed informed consent prior to participation during the first visit were included in the current analyses. Age during inclusion was calculated and used for all analyses. Self-reported data on COVID-19 were derived from 27 consecutive measurements collected in the Lifelines COVID-19 cohort study in the form of surveys between March 2020 and March 2022. A positive COVID-19 diagnosis was based on the answers to the questions on whether participants reported that they have or had SARS-CoV-2 infections and whether the doctor had told them they have (or had) a SARS-CoV-2 infection. Vaccination status was also based on self-reporting. The long COVID symptoms of headache, anosmia, memory, and concentration problems were assessed using an ordinal five-point Likert scale to identify to what extent participants were bothered by these symptoms (1 = not at all, 2 = a little bit, 3 = somewhat, 4 = quite a lot, 5 = very much) in the past seven days. The timeframe of 7 days was changed to the past 14 days when questionnaires were administered every 2 weeks and monthly. Data on headache and anosmia symptoms were obtained from 26 questionnaires in total, while data on concentration and memory problems were obtained from the last five questionnaires only.

Migraine diagnoses were self-reported based on questionnaires sent during baseline and four follow-up questionnaires until 2021. Individuals who reported suffering from migraine during at least one questionnaire were categorized as migraine patients. Migraine diagnoses that occurred after COVID-19 were not included in the analysis. The migraine status was solely based on participants' responses in the questionnaire and was not further verified by medical professionals.

The body mass index (BMI) was calculated using the standard formula of [weight (kg)/length^2^ (m)]. Weight and height were measured using a stadiometer and standard weighing scale, respectively. Both weight and height were measured without shoes, and weight was measured wearing light clothing with empty pockets.

Ever use of hormones is a composite variable for women only and based on the self-reported answers to questions on whether hormonal contraception was ever used and whether they ever received hormone therapy for perimenopausal complaints. Smoking status (current, ever, or never) was also based on self-reported answers.

Total activity scores of the validated Short QUestionnaire to ASsess Health-enhancing physical activity (SQUASH) questionnaire were used to provide an indication of weekly habitual physical activity (30, 31). The activity score (total score per week) was calculated by multiplying minutes per week by a factor for intensity. The Lifelines Diet Score, a relative measure of overall diet quality, is derived from a 110-item food frequency questionnaire and is based on the intake of food groups with positive and negative health effects (32). The intake of these food groups was expressed in grams/1,000 kilocalories, with a higher score indicating a higher diet quality. Alcohol intake was self-reported and expressed as grams per day. For these lifestyle proxies, only single scores per patient were taken into account, obtained prior to their SARS-CoV-2 infection.

Ethnicity was self-reported and reported as categories (White/East and West European, White/Mediterranean or Arabic, Black/Negroid, Asian or other—mainly including participants with a multiethnic background) based on answers to the question of which of these populations participants consider themselves belonging to. No education, primary education, lower or preparatory secondary vocational education, and junior general secondary education are categorized as “low;” secondary vocational education or work-based learning pathway and senior general/pre-university secondary education are categorized as “middle;” higher vocational education and university education are categorized as “high.”

We only included participants whose data on sex, age during baseline, and migraine status were complete. No additional exclusion criteria were applied.

### Statistical analyses

Baseline characteristics are displayed as means (standard deviation) for normally distributed data or medians [interquartile range, Q3–Q1] for non-normally distributed data, or as numbers (percentage) to express frequencies. The Anderson-Darling normality test was used to assess normality. Missing data on ethnicity, educational attainment, smoking status, BMI, alcohol intake, SQUASH activity score, and Lifelines diet score were imputed with 5 imputations and 10 iterations using the “mice” package in R. The missing data pattern was assumed to be missing at random.

Logistic regression analyses were conducted to study the association between lifetime migraine diagnoses and SARS-CoV-2 infections. These models were run on the combined imputed datasets. We report unadjusted and adjusted odds ratios (OR) with accompanying 95% Confidence Intervals (CI). We adjusted for age, sex, Lifelines diet score, educational attainment (as an indication of socioeconomic status), SQUASH activity score, and smoking status (ever smoking), as we considered the factors as potential confounding factors. Indeed, age, sex, and socioeconomic status have been shown to have a profound influence on the burden and prevalence of both migraine (33–35) as well as self-reported acute and persistent COVID symptoms or health outcomes (36, 37). In addition, modifiable lifestyle factors—including smoking, diet, and physical activity—have been reported to be associated with COVID susceptibility and severity (38), and with migraine attack onset (39, 40).

Exploratory analyses were conducted on long COVID symptoms (i.e., headache, anosmia, memory, and concentration problems), and descriptive statistics (relative frequencies) were applied. For each group (SARS-CoV-2 infected yes/no and migraine diagnosis yes/no), relative frequencies of the extent to which participants had these symptoms were calculated and reported. Relative frequencies were calculated by dividing the frequency of each individual score on the Likert scale by the total number of individuals per stratified group (who completed this questionnaire).

Both logistic regression analyses and exploratory analyses on long COVID symptoms were also performed after stratifying for sex (male/female).

All statistical analyses were performed using the R (version 4.2.0, Vienna, Austria), RStudio (2022.2.0.443, Boston, MA, USA), and SPSS 28.0.1.1 (SPSS Inc., Chicago, IL, USA). The graph was created using GraphPad Prism version 8.0.1 (GraphPad Software, Boston, MA, USA).

## Results

**Figure 1:**
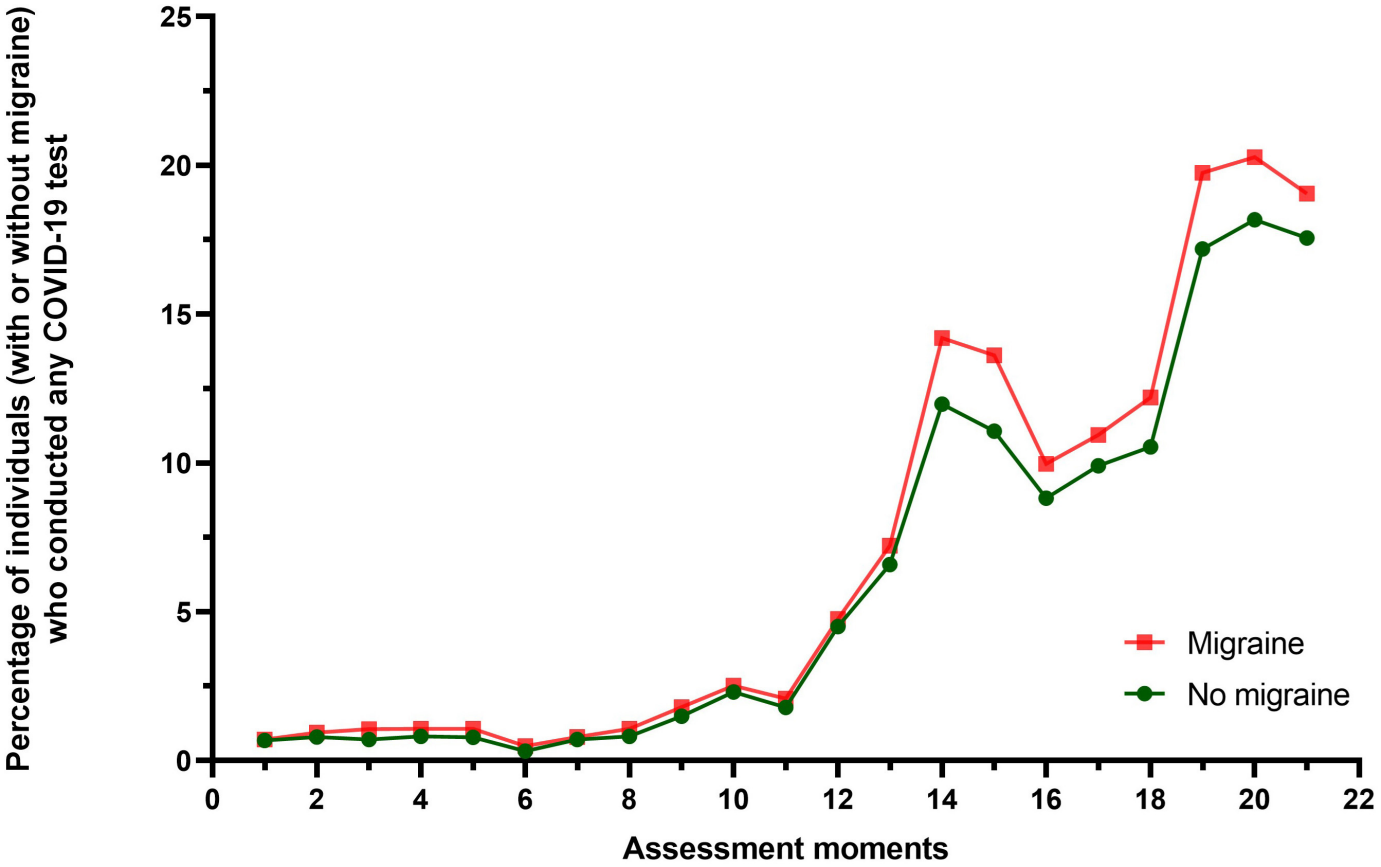
Graph illustrating the percentage of individuals with and without migraine who conducted a COVID-19 test, including both rapid tests and PCR tests, during the Lifelines longitudinal collection moments of COVID-19-related data. As the total number of individuals who did not conduct a COVID-19 test was unknown during the last six assessment moments, the percentages of these moments are not displayed.

A total of 150,507 individuals were included, consisting of 29,680 (19.7%) individuals with migraine and 120,827 (80.3%) individuals without migraine. A total of 8,664 individuals reported to have been infected with COVID-19 at least once during follow-up, consisting of 1,867 individuals with migraine (6.3% of individuals with migraine and 6,797 individuals without migraine (5.6% of individuals without migraine). The majority of individuals with migraine consisted of females (77.0% of those with migraine vs. 54.0% of those without migraine). Further, individuals with migraine were more likely to use hormonal contraception and/or hormone therapy, while individuals without migraine were more likely to be ever or current smokers and consumed more alcohol (Table 1). Notably, a total of 38,379 individuals reported to have been at least once vaccinated for COVID-19, consisting of 7,813 (20.4%) individuals with migraine and 30,566 (79.6%) individuals without migraine. While migraine patients appeared to have been tested more frequently than those without migraine, differences between these two groups were limited (Figure 1).

**Table 1:** Baseline characteristics of Lifelines participants according to self-reported lifetime migraine prevalence.

	**No migraine (*n* = 120,827)**	**Ever migraine (*n* = 29,680)**
**Female sex**, *n* (%)	65,297 (54.0%)	22,847 (77.0%)
**Age** [years], median [Q_1_-Q_3_]	44.4 [35.3–52.2]	44.0 [36.1–50.3]
**Ethnicity, n (%)**		
White/Eastern and Western European	118,396 (98.0%)	29,066 (97.9%)
White/Mediterranean or Arabic	433 (0.4%)	86 (0.3%)
Black/Negroid	189 (0.2%)	58 (0.2%)
Asian	610 (0.5%)	139 (0.5%)
Other	1,199 (1.0%)	331 (1.1%)
**Educational attainment**, n (%)		
Low	36,537 (30.2%)	8,847 (29.8%)
Middle	47,377 (39.2%)	12,520 (42.2%)
High	36,913 (30.6%)	8,313 (28.0%)
**Women who ever used hormonal contraception and/or hormone therapy**, *n* (%)	52,624 (80.6%)	18,821 (82.4%)
**Ever smoker**, *n* (%)	64,714 (53.6%)	15,574 (52.5%)
**Current smoker**, *n* (%)	25,936 (21.5%)	6,027 (20.3%)
**Body Mass Index** (kg/m^2^), median [Q_1_-Q_3_]	25.4 [23.1–28.2]	25.3 [22.9–28.4]
**Alcohol intake** [grams/days], median [Q_1_-Q_3_]	3.9 [0.8–10.3]	2.3 [0.0–6.8]
**SQUASH activity score**, median [Q_1_-Q_3_]	6,860 [4,190–10,005]	6,770 [4,230–9,690]
**Lifelines diet score**^a^, median [Q_1_-Q_3_]	24 [20–28]	24 [20–29]

^a^Lifelines diet score, calculated from quintiles of intake of positive and negative food groups in gram/1,000 kcal.

Missing values of baseline characteristics before imputation: ethnicity = 18.5%; educational attainment = 1.9%; ever smoker = 5.3%; current smoker = 5.0%; Body Mass Index = 0.08%; alcohol intake = 4.6%; SQUASH activity score = 12.0%; Lifelines diet score = 14.8%.

The adjusted odds of the probability of having SARS-CoV-2 infections were 6.3% higher among those with (a history of) migraine compared with individuals without migraine in the logistic regression model (OR = 1.06, 95% CI 1.01–1.12; Table 2). Stratification by sex showed similar results in females (OR = 1.08, 95% 1.02–1.15) but not in males (OR = 1.00, 95% CI 0.88–1.12; Table 3).

**Table 2:** The crude and adjusted association between ever having migraine and COVID-19 infections in the total study population.

	**Total population OR [95% CI]**
Unadjusted model	1.13 [1.07–1.19]
Adjusted for age, sex^*^, Lifelines dietscore, educational attainment, SQUASH activity score, ever smoking	1.06 [1.01–1.12]

^*^Females are defined as the reference group.

**Table 3:** The crude and adjusted association between ever having migraine and COVID-19 infections, stratified by sex.

	**Females OR [95% CI]**	**Males OR [95% CI]**
Unadjusted model	1.08 [1.01–1.14]	0.99 [0.87–1.11]
Adjusted for age, Lifelines dietscore, educational attainment, SQUASH activity score, ever smoking	1.08 [1.02–1.15]	1.00 [0.88–1.12]

Secondary analyses revealed that, in the total population, individuals with both migraine and COVID-19 were the most frequently bothered by headaches in the last 7–14 days, followed by those with only COVID-19 and no migraine (Table 4). Individuals without either migraine or COVID-19 experienced headaches the least frequently. While a similar pattern of reporting was observed in both sexes, females reported overall to be more bothered by headache than males (Supplementary material, Supplementary Tables 1a, 1b).

**Table 4:** The relative frequencies of the total population reporting headache in the last 7–14 days, stratified by migraine and COVID-19 status.

**Total population**	**Migraine + COVID-19 +**	**Migraine + COVID-19 –**	**Migraine – COVID-19 +**	**Migraine – COVID-19 –**
Not at all	7.59	2.63	11.45	3.64
A little bit	7.34	2.27	5.26	1.43
Somewhat	1.40	0.35	0.61	0.13
Quite a lot	0.77	0.16	0.29	0.051
Very much	0.23	0.041	0.086	0.0097

Similar results were noted for the symptom anosmia. Individuals with both migraine and COVID-19 reported the highest burden due to anosmia. They were followed by those with COVID-19 but no migraine, those with migraine but no COVID-19, and finally, those without either condition (Table 5). Yet, for anosmia, there was no discernible difference in frequency between the sexes, although females were slightly more affected by this symptom (Supplementary material, Supplementary Tables 2a, 2b).

**Table 5:** The relative frequencies of the total population reporting anosmia (loss of sense of smell or taste) in the last 7–14 days, stratified by migraine and COVID-19 status.

**Total population**	**Migraine + COVID-19 +**	**Migraine + COVID-19 –**	**Migraine- COVID-19 +**	**Migraine – COVID-19 –**
Not at all	15.98	5.25	16.56	5.087
A little bit	0.70	0.13	0.62	0.12
Somewhat	0.22	0.029	0.20	0.023
Quite a lot	0.17	0.017	0.13	0.011
Very much	0.23	0.021	0.17	0.012

The abovementioned results for memory and concentration problems were also observed across the four groups. However, being bothered by concentration problems seemed to be reported more frequently than memory problems (Tables 6, 7). Both symptoms were also reported more frequently by females across all groups (Supplementary material, Supplementary Tables 3a, 3b and Supplementary material, Supplementary Tables 4a, 4b).

**Table 6:** The relative frequencies of the total population reporting memory problems in the last 14 days, stratified by migraine and COVID-19 status.

**Total population**	**Migraine + COVID-19 +**	**Migraine + COVID-19 –**	**Migraine - COVID-19 +**	**Migraine – COVID-19 –**
Not at all	2.013	0.54	2.35	0.55
A little bit	0.57	0.12	0.46	0.099
Somewhat	0.13	0.015	0.073	0.0097
Quite a lot	0.062	0.0053	0.025	0.0027
Very much	0.015	0.0014	0.011	0.00092

**Table 7:** The relative frequencies of the total population reporting concentration problems in the last 14 days, stratified by migraine and COVID-19 status.

**Total population**	**Migraine + COVID-19 +**	**Migraine + COVID-19 –**	**Migraine- COVID-19 +**	**Migraine – COVID-19 –**
Not at all	1.88	0.53	2.27	0.55
A little bit	0.65	0.13	0.51	0.098
Somewhat	0.17	0.021	0.099	0.013
Quite a lot	0.072	0.0070	0.031	0.0035
Very much	0.017	0.0018	0.013	0.0010

## Discussion

In the population-based Dutch Lifelines cohort, we studied the interrelation between migraine and COVID-19, as well as long COVID symptoms. We observed a slightly higher prevalence of SARS-CoV-2 infections among individuals with migraine, particularly among females. Furthermore, individuals with migraine who had been infected with SARS-CoV-2—also females in particular—more frequently reported being bothered by long COVID symptoms, including headache, anosmia, and memory and concentration problems.

There are several possible explanations for the relationship between migraine patients, SARS-CoV-2 infections, and—in particular—long COVID symptoms. First, it seems likely that infections in people with migraine are more often symptomatic. This could explain why they are slightly more likely to have a positive test result, considering that they may seek testing earlier due to the more pronounced symptoms. Second, healthcare-seeking behaviors and self-report bias might have led to the increased prevalence of SARS-CoV-2 infections among migraine patients. Indeed, women with migraine, individuals with higher healthcare utilization, and those severely affected by migraine or COVID-19 are more likely to respond to surveys. Third, migraine patients might be more susceptible to coronavirus infections. Previous research suggests that increased social interactions are unlikely to be the reason for higher SARS-CoV-2 infection rates among migraine patients, as migraine patients seem to have reported higher levels of social loneliness during the lockdown (41).

Alternatively, several underlying biological mechanisms might be involved in the overlap between COVID-19 and migraine, although definitive pathophysiological mechanisms cannot be concluded from these results. One mechanism is linked to hyperresponses of mast cells and the activation of cytokine storm, including interleukin-6 and other pro-(neuro-)inflammatory markers, leading to neuroinflammatory processes, microglial activation, and peripheral sensitization (42–46). Another mechanism is linked to the overexpression of the angiotensin-converting enzyme 2, being an important target for SARS-CoV-2 entry (47–49). The trigeminal nerve has also been proposed as a site for entry for the virus into the central nervous system, potentially leading to headache symptoms and anosmia (7, 50). Recently, persistent headaches among those with both COVID-19 and migraine have been hypothesized to be due to changes in retinal nerve fiber layer thickness (51). Endothelial abnormality, being a vascular marker, has been hypothesized to underlie the pathophysiology of long COVID and is involved in migraine as well (52, 53). Furthermore, COVID-19 has been linked to hypoperfusion and changes across multiple brain networks (54)—including regions associated with cognition [e.g., dysfunction of the cingulate cortex; (55)]—while migraine itself has also been associated with cognitive impairment (56). In addition, both COVID and migraine have been associated with a decrease in serotonin levels, serving as another possible explanation for the higher frequency of reported memory and concentration problems in particular (57, 58). Furthermore, patients with pre-existing primary headaches (i.e., migraine) might be more prone to developing other secondary headaches [i.e., post-COVID headaches; (59)]. It should be noted, however, that these mechanisms become relevant only after a person has already been infected with COVID-19. Thus, they do not necessarily account for any increased susceptibility to the virus in migraine patients.

Current literature on the association between migraine and COVID-19 (symptoms) remains conflicting. Previous results from the Women's Health Study demonstrated no association between COVID-19 and migraine among older women with a history of migraine (60). However, some authors observed an association with long COVID symptoms, including a chronic headache transformation among migraine patients (15) and headaches with migraine-like features (7). We also observed that individuals with both migraine and COVID-19 were the most frequently bothered by headaches, which might be explained by the induction of certain proinflammatory cytokines and interleukins of the innate immune system (61), the aforementioned activation of the trigeminovascular system as well as structural and functional brain changes (6). However, others showed that individuals with a history of migraine who recovered from COVID-19 are more likely to experience fatigue rather than headaches as a post-COVID symptom (48). Uncertainties regarding the temporality of the onset of migraine in relation to COVID-19 and long COVID symptoms, as well as the descriptive nature of most studies, including ours, might explain these discrepancies (60). Further, the lack of an intersectional approach in some studies, disregarding differences in COVID-19 outcomes across various demographic groups and different identities, might have previously led to an underestimation of the impact of these diseases (62). Nevertheless, the clinical relevance of the observed slightly higher prevalence of SARS-CoV-2 infections among female individuals with migraine could be debated.

Despite the slightly lower number of vaccinated individuals with migraine and the marginal differences in COVID-19 testing rates, which were higher among migraine patients, we cannot exclude the possibility of a higher nocebo effect in individuals with migraine (63). Although vaccinations have been described to prevent long COVID symptoms (64), this nocebo effect might explain the increase in bothersome long COVID symptoms, including the presence of headaches (63). While the nocebo effect has, in general, been described to be larger among females (65), and might even be exaggerated by the controversy surrounding vaccination debates, we cannot exclude the possibility that this also explains the observed sex differences in long COVID symptoms.

### Strengths and limitations

Several limitations must be considered when interpreting our findings. First, we cannot exclude that the presence of sex differences is due to a lower migraine prevalence and power, but also less pronounced migraine symptomatology [including symptoms accompanying migraine headaches; (66)], among males. In addition, whether the observed sex differences can be attributed to biological factors or to behavioral attributes (i.e., gender) remains to be demonstrated (22). Second, migraine was based on self-reported questionnaires and not based on the International Classification of Headache Disorders, nor were diagnoses verified by medical professionals (67). This could have led to migraine misdiagnoses at baseline, while new daily persistent headache could have also been confused with long COVID headache due to their similar clinical characteristics (6). Further, migraine exacerbations during COVID-19 infections could be confused with secondary headaches associated with viral infection. Third, we did not differentiate between COVID-19 subtypes or mutated viruses, which could have different influences on the occurrence, severity, and duration of long COVID symptoms. Fourth, the definition of long COVID might also not have been rigorously met, as we did not clearly distinguish between “ongoing symptomatic COVID-19” (4 to 12 weeks post-infection) and “post-COVID-19 syndrome” (symptoms persisting beyond 12 weeks; 5). These phases exhibit different patterns concerning age, sex, and phenotype, which could also influence long COVID symptoms, including headache characteristics. Fifth, given that our results rely on questionnaire responses, the possibility of selection bias cannot be excluded. Asymptomatic COVID-19-infected individuals, but also more severely ill patients and those admitted to the hospital, are likely to be underrepresented in our study. We hypothesize that mainly symptomatic individuals conducted a test, although it is not unlikely that asymptomatic individuals also did—for example, if they were warned or contacted by a COVID-19-positive individual. As it is likely that the latter group is larger, we cannot rule out the possibility of information bias herein. Moreover, the correct conduct of testing procedures and accuracy of positive COVID-19 test results could not be verified due to this form of data collection. Lastly, vaccines against SARS-CoV-2 have been associated with a 2-fold increased risk of developing headaches within a week of injection, potentially due to a secondary systemic immunological reaction. However, we did not distinguish between vaccinated and non-vaccinated individuals, nor did we consider vaccination-related changes in the migraine course (68).

Notwithstanding these limitations, the large cohort size and follow-up questionnaires provided comprehensive data on migraine and several COVID-19-related outcomes while considering lifestyle factors. A key strength is our focus on sex differences, which addresses a significant gap in the existing literature and contributes to a better understanding of how sex may influence the interrelation between migraine and COVID-19-related symptoms. Indeed, both migraine and long COVID symptoms pose a major physical and mental burden on patients and healthcare services (11, 69). Future research is warranted to understand underlying mechanisms while acknowledging the potential vulnerability of migraine patients in developing such long COVID symptoms, especially when considering social and environmental factors as well as comorbidities, such as psychiatric diseases, including anxiety, post-traumatic stress disorder, and depression (70, 71). In addition, further research on SARS-CoV-2 infections is warranted in migraine patients with other chronic pain disorders and (systemic) autoimmune disorders, especially considering (i) the potential risk of SARS-CoV-2 infections to develop (incident) autoimmune diseases (72), (ii) the impact of SARS-CoV-2 infections in individuals with pre-existing or newly developed chronic pain (73) and (iii) the association between migraine, autoimmune diseases (74) and other chronic pain conditions (71). It is essential that such studies address the diagnostic challenges encountered in this study—particularly those related to self-reported migraine and COVID-19 diagnoses. Considering the fact that the Lifelines cohort consists of a relatively healthy and young population, future studies are also pivotal to extrapolate our findings to other settings.

## Conclusions

Individuals with migraine, females in particular, are slightly more likely to be infected by COVID-19. Additionally, individuals with both migraine and COVID-19 report being bothered by long COVID symptoms, including headache, anosmia, and memory and concentration problems more frequently, compared to individuals with one or none of these disease(s). Our results might suggest a shared vulnerability, susceptibility, or pathophysiology between COVID-19 and migraine, indicating the potential need for clinical surveillance of migraine patients upon recovery from SARS-CoV-2 infection. Further sex- and gender-specific research in other populations is highly recommended.

## Data Availability

The data analyzed in this study was obtained from Lifelines. Researchers can apply to use the Lifelines data used in this study. More information about how to request Lifelines data and the conditions of use can be found on their website https://www.lifelines-biobank.com/.

## Group member of Lifelines Corona Research Initiative

H. Marike Boezen^1^, Jochen O. Mierau^2,3,4^, H. Lude Franke^5^, Jackie Dekens^5,6^, Patrick Deelen^5^, Pauline Lanting^5^, Judith M. Vonk^1^, Ilja Nolte^1^, Anil P.S. Ori^5,7^, Annique Claringbould^5^, Floranne Boulogne^5^, Marjolein X.L. Dijkema^5^, Henry H. Wiersma^5^, Robert Warmerdam^5^, Soesma A. Jankipersadsing^5^, Irene van Blokland^5,8^, Geertruida H. de Bock^1^, Judith GM Rosmalen^7,9^ and Cisca Wijmenga^5^

^1^Department of Epidemiology, University of Groningen, University Medical Center Groningen, Groningen, Netherlands

^2^Department of Economics, Econometrics and Finance, Faculty of Economics and Business, University of Groningen, Groningen, Netherlands

^3^Lifelines Cohort Study and Biobank, Groningen, Netherlands

^4^Team Strategy and External Relations, University of Groningen, University Medical Center Groningen, Netherlands

^5^Department of Genetics, University of Groningen, University Medical Center Groningen, Groningen, Netherlands

^6^Center of Development and Innovation, University of Groningen, University Medical Center Groningen, Groningen, Netherlands

^7^Department of Psychiatry, University of Groningen, University Medical Center Groningen, Groningen, Netherlands

^8^Department of Cardiology, University of Groningen, University Medical Center Groningen, Groningen, Netherlands

^9^Department of Internal Medicine, University of Groningen, University Medical Center Groningen, Groningen, Netherlands
