# Supplementary material for: Sex-related differences in the association between migraine, COVID-19, and long COVID: a population-based cohort
Source: Front Neurol. 2025 Mar 21;16:1547893. doi: 10.3389/fneur.2025.1547893 (PMC11968346; doi:10.3389/fneur.2025.1547893)
Supplement: Supplementary file 1 [file Table_1.docx]

**SUPPLEMENTARY MATERIALS**

**Table 1a** – The relative frequencies of females reporting headache in the last 7-14 days, stratified by migraine and COVID-19 status

| FEMALES | Migraine +  COVID-19 + | Migraine +  COVID-19 - | Migraine -  COVID-19 + | Migraine -  COVID-19 - |
| --- | --- | --- | --- | --- |
| Not at all | 7.17 | 2.53 | 10.27 | 3.51 |
| A little bit | 7.48 | 2.37 | 5.99 | 1.75 |
| Somewhat | 1.48 | 0.38 | 0.77 | 0.17 |
| Quite a lot | 0.83 | 0.18 | 0.37 | 0.071 |
| Very much | 0.25 | 0.048 | 0.11 | 0.013 |

**Table 1b** – The relative frequencies of males reporting headache in the last 7-14 days, stratified by migraine and COVID-19 status

| MALES | Migraine +  COVID-19 + | Migraine +  COVID-19 - | Migraine -  COVID-19 + | Migraine -  COVID-19 - |
| --- | --- | --- | --- | --- |
| Not at all | 9.59 | 2.98 | 13.26 | 3.80 |
| A little bit | 6.66 | 1.94 | 4.13 | 1.073 |
| Somewhat | 1.02 | 0.26 | 0.37 | 0.075 |
| Quite a lot | 0.45 | 0.11 | 0.16 | 0.029 |
| Very much | 0.13 | 0.021 | 0.049 | 0.0055 |

**Table 2a** – The relative frequencies of females reporting anosmia (loss of sense of smell or taste) in the last 7-14 days, stratified by migraine and COVID-19 status

| FEMALES | Migraine +  COVID-19 + | Migraine +  COVID-19 - | Migraine -  COVID-19 + | Migraine -  COVID-19 - |
| --- | --- | --- | --- | --- |
| Not at all | 15.87 | 5.30 | 16.34 | 5.34 |
| A little bit | 0.69 | 0.13 | 0.63 | 0.11 |
| Somewhat | 0.22 | 0.028 | 0.21 | 0.022 |
| Quite a lot | 0.18 | 0.017 | 0.14 | 0.010 |
| Very much | 0.24 | 0.021 | 0.18 | 0.012 |

**Table 2b** – The relative frequencies of males reporting anosmia (loss of sense of smell or taste) in the last 7-14 days, stratified by migraine and COVID-19 status

| MALES | Migraine +  COVID-19 + | Migraine +  COVID-19 - | Migraine -  COVID-19 + | Migraine -  COVID-19 - |
| --- | --- | --- | --- | --- |
| Not at all | 16.48 | 5.09 | 16.90 | 4.79 |
| A little bit | 0.79 | 0.15 | 0.60 | 0.13 |
| Somewhat | 0.20 | 0.030 | 0.18 | 0.025 |
| Quite a lot | 0.16 | 0.018 | 0.12 | 0.011 |
| Very much | 0.21 | 0.023 | 0.15 | 0.012 |

**Table 3a** – The relative frequencies of females reporting memory problems in the last 14 days, stratified by migraine and COVID-19 status

| FEMALES | Migraine +  COVID-19 + | Migraine +  COVID-19 - | Migraine -  COVID-19 + | Migraine -  COVID-19 - |
| --- | --- | --- | --- | --- |
| Not at all | 1.97 | 0.54 | 2.27 | 0.57 |
| A little bit | 0.57 | 0.12 | 0.48 | 0.11 |
| Somewhat | 0.14 | 0.016 | 0.085 | 0.011 |
| Quite a lot | 0.070 | 0.0059 | 0.027 | 0.0028 |
| Very much | 0.016 | 0.0015 | 0.012 | 0.00096 |

**Table 3b** – The relative frequencies of males reporting memory problems in the last 14 days, stratified by migraine and COVID-19 status

| MALES | Migraine +  COVID-19 + | Migraine +  COVID-19 - | Migraine -  COVID-19 + | Migraine -  COVID-19 - |
| --- | --- | --- | --- | --- |
| Not at all | 2.19 | 0.54 | 2.48 | 0.53 |
| A little bit | 0.55 | 0.12 | 0.42 | 0.090 |
| Somewhat | 0.12 | 0.015 | 0.054 | 0.0084 |
| Quite a lot | 0.021 | 0.0034 | 0.020 | 0.0025 |
| Very much | 0.0092 | 0.0011 | 0.010 | 0.00087 |

**Table 4a** – The relative frequencies of females reporting concentration problems in the last 14 days, stratified by migraine and COVID-19 status

| FEMALES | Migraine +  COVID-19 + | Migraine +  COVID-19 - | Migraine -  COVID-19 + | Migraine -  COVID-19 - |
| --- | --- | --- | --- | --- |
| Not at all | 1.82 | 0.52 | 2.15 | 0.55 |
| A little bit | 0.67 | 0.14 | 0.57 | 0.11 |
| Somewhat | 0.18 | 0.021 | 0.12 | 0.015 |
| Quite a lot | 0.081 | 0.0079 | 0.034 | 0.0040 |
| Very much | 0.018 | 0.0019 | 0.014 | 0.0011 |

**Table 4b** – The relative frequencies of males reporting concentration problems in the last 14 days, stratified by migraine and COVID-19 status

| MALES | Migraine +  COVID-19 + | Migraine +  COVID-19 - | Migraine -  COVID-19 + | Migraine -  COVID-19 - |
| --- | --- | --- | --- | --- |
| Not at all | 2.14 | 0.54 | 2.45 | 0.54 |
| A little bit | 0.58 | 0.11 | 0.43 | 0.079 |
| Somewhat | 0.13 | 0.018 | 0.073 | 0.010 |
| Quite a lot | 0.028 | 0.0040 | 0.027 | 0.0029 |
| Very much | 0.0092 | 0.0014 | 0.011 | 0.00089 |
